# Flow Cytometry and T-Cell Response Monitoring after Smallpox Vaccination

**DOI:** 10.3201/eid0911.030349

**Published:** 2003-11

**Authors:** Fabrizio Poccia, Cristiana Gioia, Carla Montesano, Federico Martini, Douglas Horejsh, Concetta Castilletti, Leopoldo Paolo Pucillo, Maria Rosaria Capobianchi, Giuseppe Ippolito

**Affiliations:** * National Institute for Infectious Diseases “Lazzaro Spallanzani,” Rome, Italy

**Keywords:** Orthopoxvirus, monkeypox, cowpox, camelpox, cytomegalovirus, CMV, vaccinia, antigen, intracellular, cytokines, vaccine, exposed, flow cytometry

## Abstract

Orthopoxvirus zoonosis or smallpox as result of bioterrorism or biological warfare represents a risk for epidemic spread. By monitoring T-cell responses by flow cytometry, we observed a recall response after recent vaccination against smallpox. When the high similarity between the orthopoxviruses is considered, this rapid assay that uses vaccinia antigens could identify recently exposures.

Although the last natural case of smallpox was reported in Somalia in 1977, this orthopoxvirus remains a source of concern. No evidence exists that smallpox will recur as an endemic disease, but the virus may have been acquired for use in biological warfare or bioterrorist attacks. If one assumes an average of 15 days is needed for infected persons to become infectious, delay in intervention will be costly, increasing the total number of cases (*1*). Furthermore, the recent outbreak of the severe acute respiratory syndrome coronavirus and the first documented outbreak of monkeypoxvirus in the Western Hemisphere underline the ever-present risk for epidemic extension of zoonosis and raise concerns about the medical and social effect of reemerging orthopoxvirus infection in humans. During the epidemic spread of an emerging pathogen, evaluating exposed persons and containing the infected population should be the first priorities. A local outbreak of orthopoxvirus infection would require rapid and sensitive diagnostics, including novel assays based on host responses.

For intracellular pathogens, the antibody titers and neutralization assays represent routine immunologic tests that provide results after several weeks of infection. The appearance of a detectable antibody titer takes place a few days after the induction of a T-cell response (*2*). Moreover, antigen-specific T-cell responses could be detected in exposed, but uninfected persons, as shown in those with HIV infection (*3*). Using a rapid flow cytometric test, we previously showed that monitoring interferon (IFN)-γ production by antigen-pulsed T cells provides a quantitative and functional assessment of HIV- or cytomegalovirus (CMV)-specific CD8(+) and CD4(+) T cells (*4*–*6*). This technique requires that whole proteins or selected peptide antigens are added to blood cells, allowing the simultaneous analysis of both major histocompatibility complex class I and II restricted T-cell responses (*7*). Because smallpox vaccination was recently shown to induce a strong vaccinia virus-specific CD8(+) CTL- and IFN-γ–producing T cells detectable by more cumbersome research laboratory methods (cytotoxic, proliferative, or ELISPOT assays) (*8*,*9*), we evaluated the feasibility of an easy, rapid, and sensitive assay to monitor T-cell responses after recent vaccination against smallpox; the assay can potentially be used as a routine diagnostic assay.

## The Study

T-cell reactivity was analyzed after recent (<2 years ago) smallpox vaccinations, in long-term vaccinated (>20 years ago) and not vaccinated persons. Briefly, peripheral blood mononuclear cells (PBMC) were isolated by standard density centrifugation (Ficoll-Hypaque, Pharmacia, Uppsala, Sweden). Stimulation was also performed on whole blood samples; however, the assay had reduced sensitivity. We cannot exclude the possibility that whole bloodassay sensitivity could be improved by changing protocol conditions (data not shown). PBMC were cultured in complete Roswell Park Memorial Institute 1640 medium, 10% v/v heat-inactivated fetal calf serum, 2 mM L-glutamine, and 10 U/mL penicillin/streptomycin at a concentration of 10^6^ cells/mL. Stimulation was performed with 40 μL/mL (total protein content of approximately 1 μg/mL) of vaccinia viral antigen resuspended according to the manufacturer’s instructions (Maine Biotechnology Services, Portland, ME), or 2 μg of CMV antigen (Biowhittaker, Walkersville, MD), always in the presence of co-stimulation with both anti-CD28 and CD49d monoclonal antibodies (1 μg/mL, Becton, Dickinson and Company, Franklin Lakes, NJ). We also tested the T-cell response with live vaccinia-infected fibroblast or Vero cells. The response against uninfected antigenic preparations was always above background, reducing the sensitivity of the assay (data not shown); therefore, the commercially available antigens were used in subsequent experiments. Cultures were incubated at 37°C for 1 h, followed by an additional overnight incubation with 10 μg/mL of the secretion inhibitor Brefeldin-A (Sigma-Aldrich Corporation, St. Louis, MO). Cells were washed twice in phosphate-buffered saline, 1% bovine serum albumin, and 0.1% sodium azide, and stained for 15 min at 4°C with monoclonal antibodies specific for cell surface CD antigens (Becton, Dickinson and Company). Samples were then fixed in 1% paraformaldehyde for 10 min at 4°C, incubated with Phyco-Erithrin-conjugated mouse-anti-human IFN-γ ( (Becton, Dickinson and Company), washed twice in phosphate-buffered saline, 1% bovine serum albumin, and 0.1% saponin, and resuspended in FACSFlow before being acquired on FACScalibur (Becton, Dickinson and Company), as previously described (*4*,*6*). Controls for nonspecific staining were monitored with isotype-matched monoclonal antibodies (Becton, Dickinson and Company); cells incubated with only anti-CD28 and -CD49d were included in each experiment and nonspecific staining was always subtracted from specific results. In the cytometric panels shown in the Figure, the IFN-γ production by CD3(-) cells is 1 log lower in intensity compared to the antigen-specific CD3(+) T-cell response, representing an unspecific response that may involve natural killer cells. To monitor antigen-specific T-cell responses, we collected data only from CD3(+) T cells producing higher amounts of IFN-γ. Negative control antigenic stimulation was always below the detection limit of the assay (0.02%).

**Figure:**
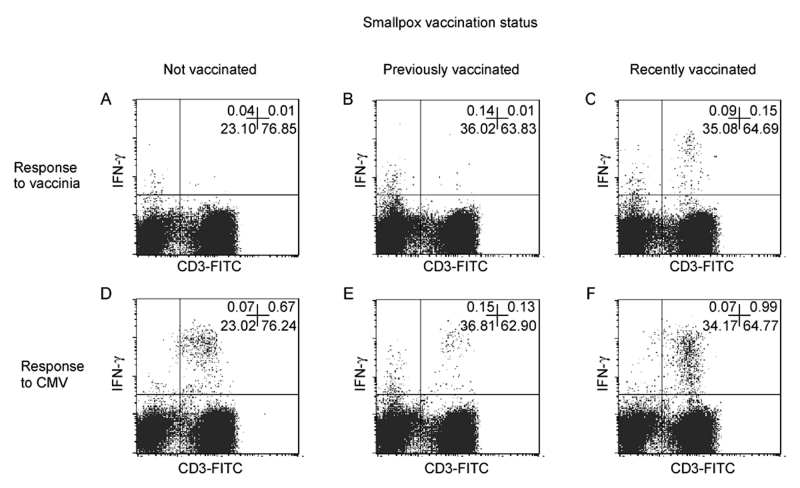
Flow cytometric analysis of T-cell responses to smallpox antigens after recent smallpox vaccination and in long-term vaccinated or not vaccinated persons. Interferon (IFN)-γ synthesis by T cells after an in vitro stimulation with vaccinia antigens was analyzed in eight healthy donors selected as recently vaccinated, long-term vaccinated, and not vaccinated persons. A representative experiment is reported in this figure. Panels A and D refer to an unvaccinated healthy donor (25-year-old white man, current neutralizing antibodies absent). A long-term vaccinated healthy person is reported in panels B and E (29-year-old white man received two doses of vaccine virus by scarification >20 years ago, current vaccinia neutralizing antibody titer of 1:8). Results from a recently vaccinated person are shown in panels C and F (31-year-old white man, single dose of Dryvax vaccine virus [Wyeth Labs, Marietta, PA] by scarification, January, 2002, current vaccinia neutralizing antibody titer of 1:32). Serum was tested for standard neutralization assay. Briefly, 0.1 mL of serial twofold dilutions of each serum was mixed with an equal volume of vaccinia virus suspension containing ~100 TCID_50_. After incubation, virus-antibody mixtures, medium, and virus controls were inoculated onto monolayers of Vero cells seeded in 96-well plates. Concomitant retitration of virus suspension was run in parallel. After 48-h incubation at 37°C, the cytopathic effect was observed under light microscope, and the microplates were stained with crystal violet. For T-cell assays, peripheral blood mononuclear cells cultures were stimulated with vaccinia virus (panels A–C) or cytomegalovirus antigens (panels D–F), and intracellular IFN-γ synthesis was analyzed in CD3(+) T cells. Percentages in panel quadrants refer to total lymphocytes.

Cytometric panels in the Figure show the IFN-γ synthesis by CD3(+) T cells after in vitro stimulation with vaccinia virus or CMV antigens. As shown in panels D, E, and F, all donors were strongly reactive to the CMV antigens (0.87%, 0.20%, and 1.53% of CD3(+) T cells respectively; the numbers of CMV-specific CD3(+) T cells per blood milliliter were 13,132, 2,964, and 11,385, respectively). As previously described (*6*), most of the CMV-specific response was related to CD4(+) T cells (96%, 75%, and 59% of CMV-specific T cells, respectively). Both unvaccinated and long-term vaccinated healthy donors had undetectable responses to smallpox vaccinia antigens (Figure, panels A and B). In contrast, a recall response was detectable after a recent immunization (Figure, panel C). In this case, the percentage of T cells specific for smallpox vaccine antigens was 0.23% among CD3(+) T cell , and the number of vaccinia antigen-specific cells was 1,725 per blood mL corresponding to a frequency of 1/667. Most vaccinia-specific T cells detected by this assay were CD4(+) (vaccinia-specific CD4(+) T cells were 80% of vaccinia-specific T cells). Nevertheless, the sensitivity of this assay to detect CD8(+) T cells could be improved by using human leukocyte antigen (HLA) class I–specific peptides as previously described (*4*).

## Conclusions

Vigorous and long-lasting protective immune responses have been associated with smallpox vaccination, and specific immunity is believed to be maintained for decades (*10*,*11*). In long-term vaccinated persons, virus-specific CD4(+) and CD8(+) T-lymphocytes are detectable only after extensive in vitro culture and restimulation to generate antigen-specific lines or clones. This limitation is due to the long, but limited, lifespan of memory T cells and to their low frequency, usually below 1/50,000 (*12*). Our in vitro rapid assay based on a short-time primary T-cell response was unable to show the residual memory T-cell response present in long-term vaccinated persons since the assay sensitivity is 1 log lower but could detect the higher frequencies of IFN-γ–producing antigen-specific cells appearing a few weeks after smallpox vaccine inoculation (*8*). Accordingly, Terajima et al. (*13*) demonstrated that T-cell responses to vaccinia and variola conserved epitopes peak 14 days after primary immunization with vaccinia virus. In this study, the frequency of antigen-specific T cells was measured as IFN-γ production by ELISPOT and HLA/peptide tetramer–staining methods. Because strong correlations between the data derived from ELISPOT, tetramer assays, and intracellular cytokine staining for IFN-γ were previously observed (*14*), vaccinia-specific T cells could be detected by flow cytometry only a few days after immunization with vaccinia virus. In addition, Pincus and Flick demonstrated the initial development of delayed hypersensitivity, an index of cell-mediated immunity, as early as 2 days after smallpox vaccination (*15*). During viral infection, high levels of virus-specific T cells are found in acute infection, falling below detectable limits as the viral load decreases and reappearing in chronic infections during episodes of transient viremia. Accordingly, we observed that the frequencies of HIV-specific CD8(+) T cells releasing IFN-γ were quantitatively increased a few weeks after viral rebound consequent to the interruption of antiviral therapy (*5*). These observations indicate that the frequency of virus-specific T cells is clinically relevant, which suggests that this method may be useful in detecting immune response by monitoring the frequency of virus-specific T cells. In recently vaccinated persons, memory cells are expanded by antigen reexposure, and their increase in frequency could be quantitatively detected by the rapid flow cytometric T-cell assay, confirming the efficacy of vaccination. Moreover, because of the high similarity between orthopoxviruses, this rapid assay using vaccinia antigens could be used to identify recently exposed persons.

Finally, an important aspect in developing a diagnostic assay is to use a rapid and easily automated system that works on virtually all persons who carry the disease. In this context, the intracellular T-cell cytokine staining by flow cytometry presents several advantages in comparison to other techniques, such as tetramer staining and ELISpot (*4*). In fact, flow cytometry allows for testing multiple proteins or peptides at a single time and provides at the same time a quantitative and phenotypic assessment of CD8(+) and CD4(+) responding T cells. Moreover, optimization of antigen preparation with peptide pools designed to be virus-specific, highly conserved, and independent of HLA haplotypes may allow for the development of a second generation of more sensitive flow cytometric T-cell assays, extending the possibility to perform routine analysis on cryopreserved samples (*4*). The technique could be easily automated through the use of analytical instruments already available in most clinical laboratories that use flow cytometry. In comparison with other analytical systems for assessing antigen-specific responses, this method is economically advantageous. The recent availability of mobile flow-cytometer units may allow use of this assay under field investigation conditions.
